# Safety and in vivo immune assessment of escalating doses of oral laquinimod in patients with RRMS

**DOI:** 10.1186/s12974-017-0945-z

**Published:** 2017-08-31

**Authors:** Tjalf Ziemssen, Hayrettin Tumani, Tony Sehr, Katja Thomas, Friedemann Paul, Nils Richter, Emil Samara, Ofer Spiegelstein, Ella Sorani, Oren Bar-Ilan, Dorit Mimrod, Liat Hayardeny

**Affiliations:** 1 Department of Neurology, MS Center Dresden, Center of Clinical Neuroscience, University Clinic Carl Gustav Carus Dresden, Dresden, Germany; 2 Multiple Sclerosis Unit, Department of Neurology, University of Ulm, Ulm, Germany; 3 Fachklinik für Neurologie Dietenbronn, Schwendi, Germany; 4 NeuroCure Clinical Research Center and Clinical and Experimental Multiple Sclerosis Research Center, Department of Neurology, Charité University Medicine, Berlin, Germany; 5 Experimental and Clinical Research Center, Max Delbrueck Center for Molecular Medicine and Charité University Medicine Berlin, Berlin, Germany; 6 Neurologische Gemeinschaftspraxis, Düsseldorf, Germany; 7 PharmaPolaris International, Davis, CA USA; 8 Teva Pharmaceutical Industries, Netanya, Israel; 9 Galmed Pharmaceuticals, Tel Aviv, Israel

**Keywords:** Laquinimod, Multiple sclerosis, Safety, Immunology, slanDC

## Abstract

**Background:**

Laquinimod is an oral immunomodulator in clinical development to treat relapsing-remitting multiple sclerosis (RRMS). Laquinimod is in clinical development for the treatment of multiple sclerosis and Huntington Disease (HD). The objective of this study is to assess the safety, tolerability, pharmacokinetics (PK) and cytoimmunologic effects following escalating doses of laquinimod in patients with RRMS.

**Methods:**

One hundred twelve patients were randomly assigned to laquinimod/placebo in a series of separate dose-escalating cohorts starting from a daily oral dose of 0.9 mg/1.2 mg escalating to 2.7 mg, in 0.3 mg increments.

**Results:**

Twenty-eight patients received placebo and 84 received laquinimod ranging from 0.9 to 2.7 mg. No deaths occurred. One serious adverse event (SAE) of perichondritis was reported, which was unrelated to laquinimod (0.9 mg). There was no increased incidence of adverse events (AEs) with escalating doses. Laquinimod-treated patients showed more abnormal laboratory levels in liver enzymes, P-amylase, C-reactive protein (CRP), and fibrinogen, but most shifts were clinically non-significant. The exposure of laquinimod was dose proportional and linear in the tested dose range. An immunological substudy showed significant dose-dependent decreases in 6-sulpho LacNAc + dendritic cell (slanDC) frequency following laquinimod compared to placebo.

**Conclusion:**

Laquinimod doses up to 2.7 mg were safely administered to patients with RRMS. An in vivo effect of laquinimod on the innate immune system was demonstrated.

**Trial registration:**

EudraCT Number: 2009-011234-99. Registered 23 June 2009.

**Electronic supplementary material:**

The online version of this article (doi:10.1186/s12974-017-0945-z) contains supplementary material, which is available to authorized users.

## Background

Laquinimod, a quinolone-3-carboxamide derivative, is an innovative oral anti-inflammatory drug selected for efficacy and safety from a pool of 60 quinoline 3-carboxamide derivatives of the parent compound roquinimex, a drug whose clinical development stopped due to safety concerns [1]. Laquinimod is in clinical development for the treatment of multiple sclerosis and Huntington disease (HD). Three phase 3 studies evaluating the efficacy and safety of laquinimod 0.6 mg as a treatment for relapsing remitting MS have been conducted; one study (Allegro) showed statistically significant differences between laquinimod and placebo in its primary endpoint of relapse rate reduction [2], the second study (BRAVO) did not [3]. The third study, Concerto did not meet its primary endpoint of a difference from placebo in confirmed 3-month disease progression; however, there were differences from placebo in the secondary endpoints [4]. There are two clinical trials currently ongoing, one evaluating laquinimod as a treatment for progressive MS [5], and one with laquinimod as a HD treatment [6].

This maximum tolerated dose study (MS-LAQ-101) was designed to assess the safety and tolerability profile of ascending doses of oral laquinimod (0.9, 1.2, 1.5, 1.8, 2.1, 2.4, and 2.7 mg) administered daily in patients with relapsing-remitting multiple sclerosis (RRMS). It is important to note that in January 2016, subsequent to the completion of the study reported herein, patients on laquinimod doses greater than 1.0 mg per day were discontinued from treatment in the clinical studies of MS and HD. This action was recommended by the Data Monitoring Committee and was due to an imbalance in cardio- and cerebrovascular adverse events (AEs) in emerging safety data in the MS clinical studies (Teva Pharmaceutical Industries Ltd., data on file). The study evaluating the effect of laquinimod as a treatment for progressive MS includes laquinimod at a dose of 0.6 mg per day as a MS treatment, and the study on laquinimod as a treatment for HD includes doses of 0.5 and 1.0 mg.

Preclinical studies have shown that laquinimod reduces inflammatory cell infiltrates in the central nervous system (CNS), inhibits development of experimental autoimmune encephalomyelitis, decreases demyelination, and prevents axonal loss [7, 8], and the formation of meningeal B cell aggregates [9]. Laquinimod is likely to exert its anti-inflammatory activity via suppression of Th1 and Th17 cells and induction of a Th2/3 shift of immune response in the peripheral blood [7–13]. These changes are mainly related to changes of dendritic cell properties [8, 14, 15]. A possible mechanism of laquinimod associated neuroprotective activity was demonstrated in the cuprizone model of toxic demyelination [16] where laquinimod treatment prevented cuprizone-induced demyelination by attenuating astrocytic NF_Ƙ_B activation in a mechanism involving direct CNS intrinsic modulation of NF_Ƙ_B signaling in astrocyte [17] independent of Toll-like receptor signaling [18]. These results were further verified using human astrocytes in vitro [17]. Laquinimod’s immunomodulatory effects in EAE are thought to be mediated via its action at the aryl hydrocarbon receptor (AhR) [19].

Previously in a longitudinal analysis of immune parameters comparing laquinimod and placebo-treated cohorts, no significant changes in the relative proportion of T cells, B cells, monocytes and macrophages, natural killer cells, dendritic cells, or FoxP3^+^ CD25^hi^ regulatory T cells were observed in patients, receiving 2 years of continuous laquinimod therapy and they retained their capacity to respond to immunologic stimuli [20]. However, detailed in vitro analysis of monocytes demonstrated a lower level of CD86 expression on monocytes stimulated with lipopolysaccharide (LPS) in laquinimod patients starting from the first month of treatment. Upon inflammatory stimulation, monocytes obtained from laquinimod-treated patients tended to secrete lower levels of the proinflammatory chemokines CCL2 or CCL5 [21]. As existing data suggest that inhibition of the NF-κB pathway is responsible for the changes observed in dendritic cell maturation and functions, laquinimod may exhibit its disease-modulating activity in MS by downregulating immunogenicity of dendritic cell responses [22]. A recent study has showed the proinflammatory potential of 6-sulpho LacNAc^+^ dendritic cells (slanDCs) through expression of CD83 and tumor necrosis factor-α (TNF-α) production, and that a decrease in slanDCs was linked to decreased inflammatory activity [23]. Thus, as part of this study to assess the safety and tolerability of ascending laquinimod doses, the effect of laquinimod on the frequency of slanDCs, a cell population of interest that accounts for 0.5 to 2% of PBMCs and have been recently described in MS, was evaluated [24, 25].

## Methods

### Participants and study design

This phase 1, randomized, double-blind, placebo-controlled, dose-escalation study was performed at seven centers in Germany from August 2009 to March 2012. The study was conducted in accordance with the Declaration of Helsinki, the protocol was approved by the Independent Ethics Committee at each study site (Ethikkommission der Universität Ulm), and written informed consent was obtained from each patient as a condition of entry. Patients participating in the immunological substudy were required to sign a separate informed consent form. Eligible participants were male and female patients aged 18 to 55 years old, inclusive, with a confirmed and documented diagnosis of RRMS (revised McDonald criteria) [26], had experienced at least one documented relapse in the 3 years prior to screening, and were ambulatory with a baseline EDSS score [27] of 0 to 5.5.

This study assessed the tolerability and safety of escalating doses of oral laquinimod (0.9, 1.2, 1.5, 1.8, 2.1, 2.4, and 2.7 mg) administered daily for 4 weeks in patients with RRMS (see Additional file 1: Figure S1 for study design). As there was no formal hypothesis testing planned, no statistical methods were employed to determine sample size. Based upon clinical judgment, 16 patients per cohort (randomly assigned in a 3:1 ratio for laquinimod and placebo, respectively), was considered adequate for this type of study. An exception was the first cohort, which consisted of two laquinimod arms (0.9 and 1.2 mg) and a placebo arm, with 32 patients randomly assigned in a 3:3:2 ratio. The Teva Global Biostatistics unit prepared a computer-generated randomization scheme for each cohort using a SAS® PLAN procedure. Each scheme used a block design; however, due to the small number of patients recruited at each center for each cohort, there was no stratification by center.

The investigator at each study site evaluated the eligibility of patients to participate in the study during a 1-week screening and baseline visit. At the baseline visit, patients who met inclusion criteria were randomized to receive either laquinimod or matching placebo capsules by an Interactive Web Response System according to the randomization algorithm. Laquinimod capsules and their matching placebo capsules were of identical appearance and packaged in aluminum-silver/aluminum-soft blister cards to maintain study blinding. All patients were administered laquinimod or matching placebo capsules, taken at the same hour every day, with water.

The investigators, the sponsor, and any personnel involved in patient assessment, monitoring, analysis, and data management (excluding the designated Clinical Supply Chain’s personnel) were blinded to patient assignment. Once each dose cohort was completed and the database closed, treatment assignments of that cohort were unblinded to the sponsor study team to allow for further data analysis, presentation to the steering committee (SC), and a decision whether to continue to the next dose cohort in 0.3 mg increments of laquinimod. The investigators remained blinded to the patients’ treatment assignment. Specific predefined safety stopping rules relating to increases in alanine transaminase (ALT) or aspartate transaminase (AST) ≥ 3 × the upper limit of normal were included in this study. At any time during the study, the SC could determine if a dose-limiting toxicity (DLT) had occurred. The criteria for DLT were not predefined and were based on the judgment of the SC.

Scheduled in-clinic visits during which clinical examinations and safety evaluations were performed occurred at screening (day −7), baseline (day 0), and on days 7, 14, 21, and 28. Drug treatment discontinued on day 28 (end of double-blind treatment visit) and a 2-week follow-up (study completion) visit occurred on day 42. Patients who discontinued study drug prior to the day 28 visit performed the follow-up/study completion visit 14 days after study drug discontinuation. Unscheduled visits for safety or any other reason occurred as needed during the study.

Blood samples for pharmacokinetic (PK) evaluation were collected on day 21 before dosing, and at 0.25, 0.5, 1, 2, 3, 4, 6, and 24 h after dosing. In addition, predose samples were collected on days 7, 14, and 28. Samples were analyzed for laquinimod concentrations by LC/MS/MS bioanalysis assay using validated methods with a lower limit of quantification of 2.5 ng/mL. The pharmacokinetic measures were evaluated by model-independent methods using Phoenix WinNonlin version 6.2. Actual sampling times were used for pharmacokinetic analysis, while nominal sampling times were used for summary statistics. Further details regarding preparation of PBMC samples are provided in the Additional file 1.

As part of the immunological substudy, whole blood samples for assessment of PBMCs were collected on days 0 and 28 from all patients who signed the appropriate informed consent form. Whole blood samples for detailed longitudinal assessment of PBMCs at the Dresden study center were also collected for evaluation on days 7, 14, 21, and 42. SlanDCs were evaluated regarding frequency, properties, and modulating effects of laquinimod on activation status.

### Measures and statistical analyses

This study aimed to assess the safety and tolerability profile of ascending doses of laquinimod administered daily in patients with RRMS. Safety assessments included evaluation of adverse events (AEs), clinical laboratory (biochemistry, hematology, and urinalysis) assessments, vital signs, and electrocardiograms (ECGs). The intent-to-treat analysis set (ITT) consisted of all patients randomized to the study and who received at least one dose of study drug. In accordance with the ITT principle, all patients were kept in their originally randomly assigned treatment group. All safety analyses were performed on the ITT analysis set.

For each of the study doses, the following steady-state PK parameters were derived using noncompartmental methods: maximum plasma concentration (*C*_max_), minimum plasma concentration (*C*_min_), time to maximum plasma concentration (*t*_max_), and area under the plasma concentration-time curve from 0 to 24 h postdose (AUC_0–24_).

The safety and immunological substudy data were described using descriptive statistics. ANCOVA analyses of the dose proportionality of the PK measures were performed using a linear regression model with natural logarithm of dose normalized *C*_max_ and AUC_0–24_ on day 21 as response variable (*Y*) and dose (Dose) as an explanatory variable (SAS Institute; version 9.2). Gender and body weight were included as covariates in the model. A linear model *Y* = *a* + *b* × Dose + error was used to fit the data and construct 95% confidence intervals (CI) for *b*. The PK parameter was declared dose proportional if the slope parameter *b* was not significantly different from 0 or the 95% CI for *b* contained 0.

## Results

### Study population

**Fig. 1:**
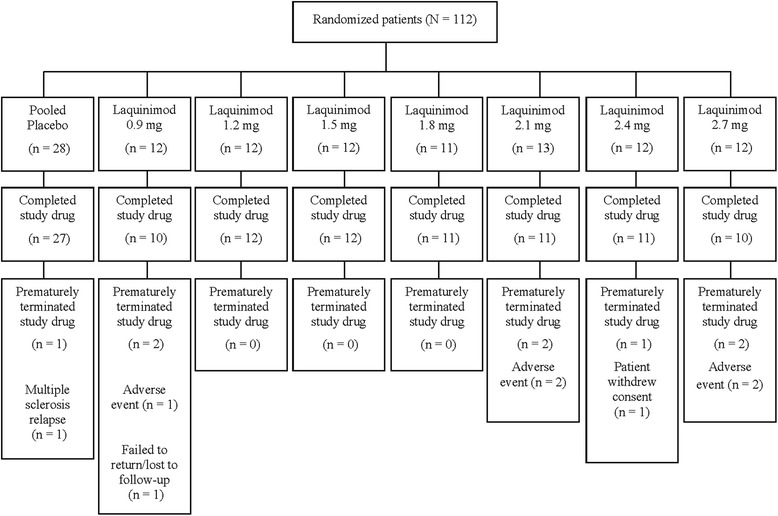
Patient disposition. All patients enrolled in the study were included in the safety evaluation. All safety analyses were performed on the ITT analysis set. ITT intent-to-treat

A total of 112 patients participated in the study (Table 1). Twenty-eight patients (23 females, 5 males) received placebo, and 84 patients (56 females, 28 males) received daily doses of laquinimod ranging between 0.9 and 2.7 mg (Fig. 1). Seven patients on laquinimod and one patient on placebo terminated the study early; reasons for withdrawal in the laquinimod group included AE (*n* = 5), patient withdrawal of consent (*n* = 1), and lost to follow-up (*n* = 1) and in the placebo group MS relapse (*n* = 1). All patients enrolled in the study were included in the safety evaluation.

**Table 1:** Patient demographic and disease characteristics at baseline

MS-LAQ-101	Pooled placebo (*n* = 28)	Laquinimod 0.9 mg (*n* = 12)	Laquinimod 1.2 mg (*n* = 12)	Laquinimod 1.5 mg (*n* = 12)	Laquinimod 1.8 mg (*n* = 11)	Laquinimod 2.1 mg (*n* = 13)	Laquinimod 2.4 mg (*n* = 12)	Laquinimod 2.7 mg (*n* = 12)	All (*N* = 112)
Age, years									
Mean ± SD	37.3 ± 8.5	38.6 ± 10.3	35.8 ± 9.4	35.2 ± 5.3	35.5 ± 8.4	41.5 ± 9.8	38.3 ± 10.5	44.8 ± 7.5	38.3 ± 9.0
Range	20.9–53.9	21.4–55.9	18.6–48.4	28.8–44.8	23.2–47.2	23.4–56.0	23.6–54.5	31.3–55.5	18.6–56.0
Gender *N* (%)									
Female	23 (82.1)	10 (83.3)	6 (50.0)	10 (83.3)	5 (45.5)	7 (53.8)	9 (75.0)	9 (75.0)	79 (70.5)
Male	5 (17.9)	2 (16.7)	6 (50.0)	2 (16.7)	6 (54.5)	6 (46.2)	3 (25.0)	3 (25.0)	33 (29.5)
Height (cm)									
Mean ± SD	170.3 ± 9.5	174.2 ± 9.8	175.8 ± 8.5	169.6 ± 8.1	177.7 ± 10.5	171.9 ± 7.9	171.4 ± 7.7	173.1 ± 6.8	172.6 ± 8.9
Range	157.0–196.0	158.0–198.0	164.0–196.0	158.0–180.0	158.0–193.0	158.0–186.5	161.0–186.0	164.0–187.0	157.0–198.0
Weight (kg)									
Mean ± SD	72.9 ± 15.3	74.8 ± 14.5	75.9 ± 17.4	77.7 ± 19.1	81.1 ± 18.6	81.0 ± 13.9	79.9 ± 9.9	77.4 ± 12	76.9 ± 15.2
Range	53.0–110.0	57.0–99.5	58.0–124.0	52.0–113.0	63.0–123.2	62.0–109.5	62.0–98.0	62.0–97.0	52.0–124.0
BMI (kg/m^2^)									
Mean ± SD	24.9 ± 3.7	24.8 ± 5.5	24.4 ± 4.3	27.0 ± 6.4	25.7 ± 5.0	27.4 ± 4.2	27.3 ± 3.9	25.8 ± 3.6	25.8 ± 4.5
Range	20.2–36.6	19.8–39.9	18.9–32.3	18.6–36.5	19.1–35.8	21.1–33.0	22.0–33.9	21.5–33.6	18.6–39.9
EDSS score at screening									
Mean ± SD	2.1 ± 1.1	2.0 ± 1.4	1.6 ± 1.2	2.1 ± 1.3	2.4 ± 1.8	2.5 ± 1.2	2.7 ± 1.0	2.6 ± 1.5	–
Range	0.0–5.5	0.5–5.5	0.0–4.0	0.0–5.5	0.0–5.5	1.0–5.0	1.0–4.0	1.0–5.5	–
Total number of exacerbations in the year prior to screening									
Mean ± SD	0.7 ± 0.6	1.2 ± 0.8	1.1 ± 0.8	0.8 ± 0.7	1.2 ± 1.2	1.0 ± 0.9	1.0 ± 0.6	1.0 ± 0.6	0.9 ± 0.8
Range	0.0–2.0	0.0–3.0	0.0–3.0	0.0–2.0	0.0–4.0	0.0–3.0	0.0–2.0	0.0–2.0	0.0–4.0

*BMI* body mass index, *EDSS* Expanded Disability Status Scale, *SD* standard deviation

### Safety and tolerability

No deaths occurred during the study. One laquinimod 0.9 mg-treated patient had a serious adverse event (SAE) of perichondritis considered unrelated to study drug. In addition, one patient in the placebo group for laquinimod 1.8 mg had two SAEs of fall and skin laceration. Although there was no overall increased incidence of AEs with increased dose, several AEs occurred more frequently in the higher dose groups than in the other dose groups without a clear dose response (Table 2). The most commonly reported AE in the laquinimod groups was headache, exhibiting no clear dose response (Table 2). There was a single AE of tension headache in the 2.7 mg group considered severe.

**Table 2:** Adverse events

MS-LAQ-101 % of patients Preferred term	Pooled placebo (*n* = 28)	Laquinimod 0.9 mg (*n* = 12)	Laquinimod 1.2 mg (*n* = 12)	Laquinimod 1.5 mg (*n* = 12)	Laquinimod 1.8 mg (*n* = 11)	Laquinimod 2.1 mg (*n* = 13)	Laquinimod 2.4 mg (*n* = 12)	Laquinimod 2.7 mg (*n* = 12)
All	96.4	75	100	83.3	90.9	92.3	100	100
Headache	32.1	50	66.7	41.7	18.2	38.5	91.7	41.7
C-reactive protein increased	7.1	16.7	16.7	8.3	0	7.7	8.3	33.3
Vomiting	0	0	0	8.3	0	7.7	0	33.3
Abdominal pain upper	10.7	0	16.7	8.3	9.1	7.7	33.3	25
Back pain	0	16.7	0	16.7	0	7.7	25	25
Blood fibrinogen increased	0	0	16.7	0	0	7.7	0	25
Myalgia	0	0	0	0	9.1	0	8.3	25
Nausea	7.1	8.3	0	8.3	0	7.7	25	25
Tension headache	0	0	0	0	0	0	0	25
Diarrhea	7.1	8.3	8.3	8.3	9.1	15.4	8.3	16.7
Insomnia	0	0	0	0	0	0	8.3	16.7
Blood creatine Phosphokinase increased	14.3	0	8.3	8.3	18.2	0	0	8.3
Nasopharyngitis	10.7	16.7	33.3	8.3	0	38.5	8.3	8.3
Oropharyngeal pain	0	0	0	0	18.2	0	0	8.3
Pain in extremity	0	8.3	8.3	0	18.2	0	0	8.3
Dizziness	10.7	0	8.3	0	9.1	0	25	0
Contusion	0	0	0	0	0	7.7	16.7	0

Common AEs: AEs reported by at least two patients in any of the laquinimod dose groups and with an incidence higher than the pooled placebo

Five laquinimod-treated patients terminated early due to AEs. One patient in the 0.9 mg group terminated early due to an AE of mild headache and one patient in the laquinimod 2.1 mg group terminated due to asthenia, upper abdominal pain, and chest pain, an ECG performed 2 days later was normal. A second patient in the laquinimod 2.1 mg group terminated due to self-reported “hypersensitivity” with related chest discomfort. At the time of early termination, no allergic reaction could be determined, ECG was normal and fibrinogen level increased to 4.88 g/L (normal range 1.5–4.3 g/L, level at baseline was 4.11 g/L). At a follow-up visit 2 weeks after termination, ECG showed mild tachycardia and fibrinogen level normalized (3.68 g/L). Two patients in the laquinimod 2.7 mg group terminated the study early due to AEs. The first patient terminated due to vomiting, nausea, and tension headache. The second patient was terminated due to lab abnormalities (C-reactive protein (CRP) increased, blood fibrinogen increased). One patient in the placebo group for the 2.4 mg dose terminated the study early due to an MS relapse. No dose response detected in the incidence of AEs leading to ET and no specific AE identified as a common cause for ET across cohorts.

Overall, most patients had within normal range values for all laboratory parameters assessed throughout the study. No clear dose response observed for central trends or post-baseline shifts for any laboratory parameter. During the study, no patient had an abnormal ECG reading considered clinically significant by the investigator; a complete distribution of the investigator’s ECG interpretation per treatment group at each study visit is shown in Additional file 1: Table S1. With the limitations of small sample size, no clear dose responses were observed in the changes of mean group levels over time in any of the biochemical parameters (Additional file 1: Table S2). Laquinimod-treated groups had a higher incidence of post-baseline shifts to abnormally high CRP levels (most were not potentially clinically significant (PCS)) and to abnormally high fibrinogen levels (none were PCS). Laquinimod-treated groups also had a higher incidence of post-baseline shifts to abnormally high AST, ALT, and gamma glutamyl transferase (most non-PCS).

No specific trend of change from baseline over time seen for any hematological parameter in any of the groups (Additional file 1: Table S3). The only post-baseline shift to PCS values, detected with a higher incidence in the laquinimod arms than in the pooled placebo, was a change from high/non-PCS to low PCS hemoglobin, observed for 1 patient each at the 2.1 and 2.4 mg doses but not at the 2.7 mg dose.

No specific trend of change from baseline over time detected in any of the vital signs parameters. No dose response in the incidence of post-baseline shifts of vital signs and no patient had an abnormal ECG reading considered clinically significant by the investigator.

### Pharmacokinetics

The PK population comprised 81 patients with 10 to 12 patients per dose group. After oral administration of laquinimod, plasma concentrations reached maximum levels in the majority of patients within 2 h after dosing. Plasma concentrations declined slightly over the 24-h dosing interval, a reflection of the long terminal half-life of laquinimod (approximately 80 h). The plasma concentrations generally increased with dose (Additional file 1: Figure S2), and thus PK parameters, *C*_max_ and AUC_0–24,_ generally increased with dose (Table 3). Dose-normalized *C*_max_ and AUC_0–24_ values were comparable across all dosing groups (Additional file 1: Figure S3). The systemic exposure of laquinimod was dose proportional in the 0.9 to 2.7 mg range, as slope parameters for the linear regression model were not significantly different from 0 and 95% CIs for dose-normalized *C*_max_ and AUC_0–24_ included 0 (C_max_, 95% CI −0.056, 0.113; *p* = 0.5096 and AUC_0–24,_ 95% CI −0.068, 0.113; *p* = 0.6278).

**Table 3:** Pharmacokinetic parameters (mean ± SD) of laquinimod in MS patients following repeated daily administration for 21 days

Dose (mg)	*n*	*t*_max_^a^ (h)	*C*_max_ (ng/mL)	*C*_min_ (ng/mL)	AUC_0–24_ (ng h/mL)	Dose -normalized	
						*C*_max_	AUC_0–24h_
0.9	10	0.99 (0.25–2.00)	639.6 ± 114.9	469.5 ± 103.4	12,245.8 ± 2075.4	710.6 ± 127.6	13,606.5 ± 2306.0
1.2	12	0.75 (0.25–4.00)	779.2 ± 115.7	565.7 ± 121.0	15,075.2 ± 2757.6	649.4 ± 96.4	12,562.7 ± 2298.0
1.5	12	1.00 (0.50–24.00)	1207.8 ± 210.8	904.0 ± 189.5	22,974.0 ± 3756.6	805.2 ± 140.6	15,316.0 ± 2504.4
1.8	11	0.50 (0.25–3.00)	1449.4 ± 325.4	1076.7 ± 274.3	28,042.7 ± 6495.5	805.2 ± 180.8	15,579.3 ± 3608.6
2.1	11	2.00 (0.50–6.00)	1712.1 ± 385.8	1310.5 ± 356.0	33,741.2 ± 8133.4	815.3 ± 183.7	16,067.2 ± 3873.1
2.4	10	0.50 (0.50–2.00)	1892.3 ± 526.3	1389.3 ± 512.9	35,470.0 ± 11,100.2	788.5 ± 219.3	14,779.2 ± 4625.1
2.7	10	1.00 (0.5–2.00)	1826.2 ± 313.4	1364.5 ± 186.7	35,274.5 ± 5973.0	676.4 ± 116.1	13,064.6 ± 2212.2

*AUC*_*0–24*_ area under the plasma concentration-time curve from 0 to 24 h postdose, *C*_*max*_ maximum plasma concentration, *C*_*min*_ minimum plasma concentration, *t*_*max*_ time to maximum plasma concentration

^a^Median (range) is provided for *t*_max_

Predose plasma concentrations measured throughout the study increased with increasing doses and seemed to be comparable for days 14, 21, 22, and 28 across all dose groups, suggesting steady state was attained after approximately 14 days of daily dosing, consistent with the elimination half-life of laquinimod. Laquinimod was rapidly absorbed after oral administration and eliminated slowly from the circulation as suggested by the small differences between *C*_max_ and *C*_min_.

### Immunological substudy

**Fig. 2:**
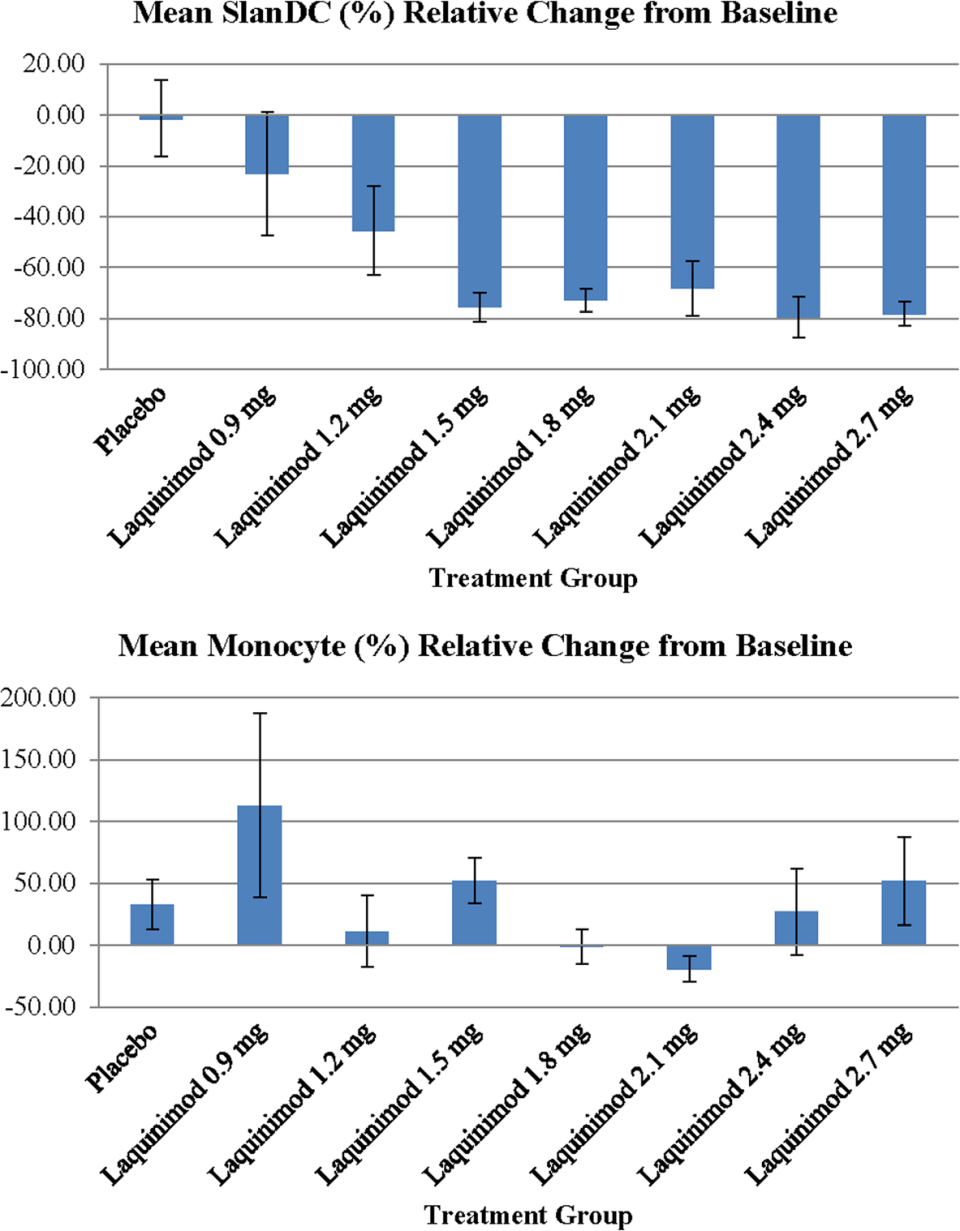
Dose-dependent effect of laquinimod (LAQ) on the frequency of monocytes and slanDC. Sample sizes for slanDC (%) relative change from baseline: placebo *n* = 20, 0.9 mg LAQ *n* = 4, 1.2 mg LAQ *n* = 6, 1.5 mg LAQ *n* = 9, 1.8 mg LAQ *n* = 7, 2.1 mg LAQ *n* = 9, 2.4 mg LAQ *n* = 8, 2.7 mg LAQ *n* = 6. Sample sizes for monocyte (%) relative change from baseline: placebo *n* = 20, 0.9 mg LAQ *n* = 4, 1.2 mg LAQ *n* = 6, 1.5 mg LAQ *n* = 9, 1.8 mg LAQ *n* = 9, 2.1 mg LAQ *n* = 10, 2.4 mg LAQ *n* = 10, 2.7 mg LAQ *n* = 8. BL baseline, fm from, LAQ laquinimod, Rel. relative, slanDC 6-sulpho LacNAc^+^ dendritic cell

A dose-dependent, in vivo effect of laquinimod on the innate immune system was demonstrated. While monocyte frequency was not affected, dose-dependent decreases in slanDC frequency were observed in the laquinimod group, but not in the placebo group. A saturation effect was found at a laquinimod dose of 1.5 mg; a further increase of laquinimod dose did not result in additional decreases in slanDC frequency in peripheral blood (Fig. 2).

**Fig. 3:**
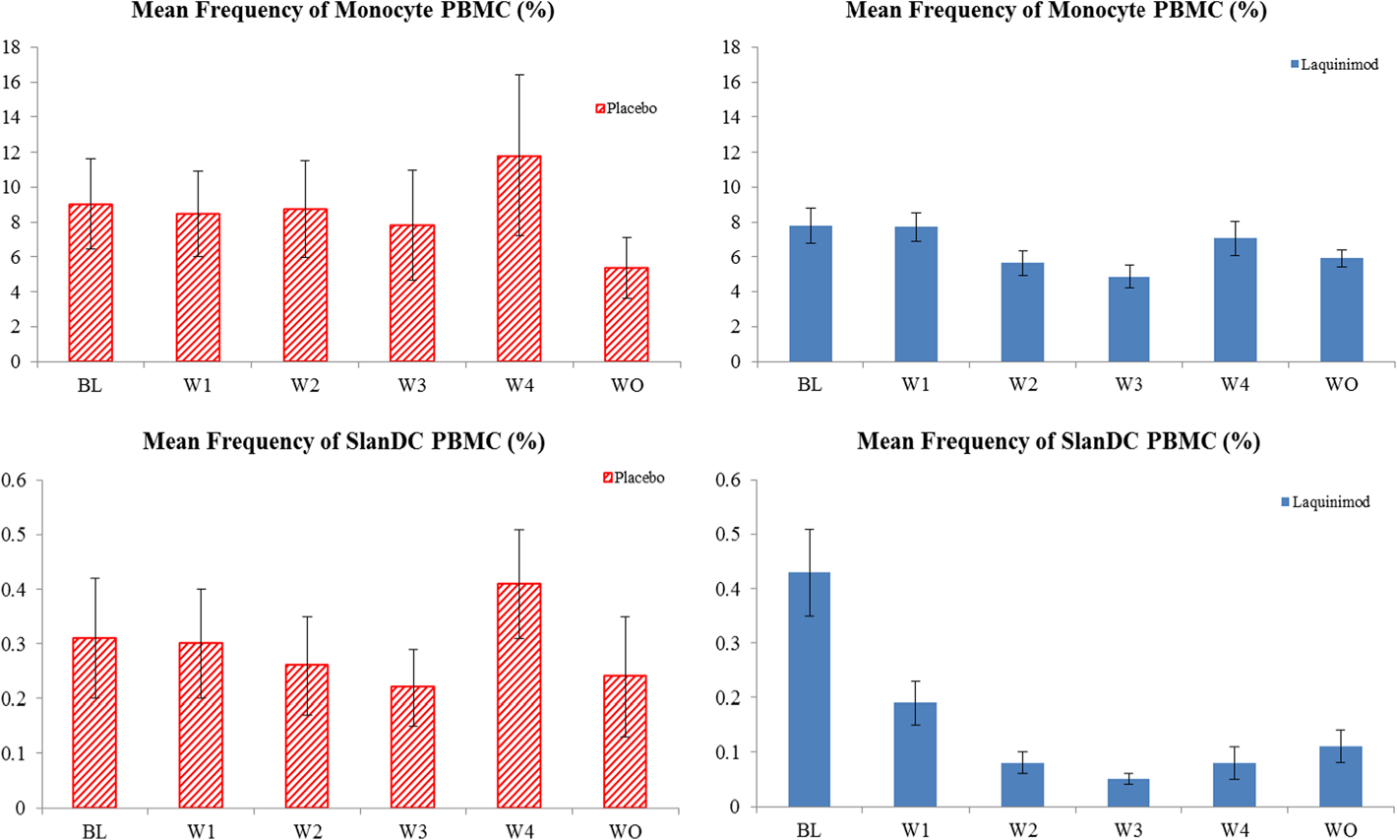
Longitudinal effect of laquinimod (LAQ) on slanDC and monocytes. Sample sizes for placebo *n* = 5, sample sizes for laquinimod *n* = 12. BL baseline, Freq frequency, PBMC peripheral blood mononuclear cell, slanDC 6-sulpho LacNAc^+^ dendritic cell, W week, WO washout

This decrease in slanDC frequency occurred very early within the first week of laquinimod treatment and reached maximum depletion after 2 weeks. Following cessation of laquinimod treatment on day 28, slanDC depletion showed a trend of recovery (Fig. 3) in day 42 (washout) samples. When compared to placebo, the proinflammatory capacity of slanDCs for CD83 expression and TNF-α production after in vitro stimulation with LPS or R848 decreased with laquinimod therapy (Table 4). The decrease from baseline in CD83 expression in slanDCs culture after LPS stimulation was −2.34 and −18.79%, and after R848 stimulation −5.55 and −16.52% for placebo and laquinimod groups, respectively. The decrease from baseline in TNF expression after LPS stimulation was −2.99 and −9.38%, and after R848 stimulation, −2.75 and −8.92% for placebo and laquinimod groups, respectively.

**Table 4:** Modulating effects of laquinimod on activation status of slanDC

	Placebo			Laquinimod		
Parameter	*n*	Mean	Std Err	*n*	Mean	Std Err
SlanDC CD83-culture LPS (%) difference from baseline	13	−2.3	5.6	31	−18.8	4.2
SlanDC CD83-culture R848 (%) difference from baseline	13	−5.5	6.4	31	−16.5	3.2
SlanDC TNF-culture LPS (%) difference from baseline	15	−3.0	3.2	37	−9.4	2.5
SlanDC TNF-culture R848 (%) difference from baseline	15	−2.3	3.1	37	−8.9	2.3

*LPS* lipopolysaccharide, *slanDC* 6-sulpho LacNAc + dendritic cell, *Std Err* standard error, *TNF* tumor necrosis factor

## Discussion

In this 4-week dose-escalating study of laquinimod for doses ranging from .9 to 2.7 mg, no data suggestive of a dose-dependent safety signal were identified. The overall incidence of AEs was comparable between the laquinimod and placebo groups; however, several AEs occurred more frequently in the higher laquinimod doses than in the other groups without a clear dose response. The AE reported as common in all laquinimod groups was headache, exhibiting no clear dose response or typical pattern. Although the shifts were not considered potentially clinically significant (PCS), the laquinimod-treated groups had a higher incidence of post-baseline shifts in some biochemical and hematological parameters (CRP, fibrinogen, AST, ALT, gamma glutamyl transferase, and hemoglobin). No safety signals detected in urinalysis, vital signs, or ECG parameters. The exposure of laquinimod was dose proportional and linear in the dose range of 0.9 to 2.7 mg. Laquinimod was rapidly absorbed after oral administration and steady state attained within approximately 14 days of dosing.

SlanDC seem to serve as an immunological marker regarding dose effects [23]. In the immunological substudy, dose-dependent decreases in slanDC frequency were observed in the laquinimod group but not in the placebo group. The relationship between the reduction in SlanDC and AhR activation by laquinimod is unknown. Monocyte frequency was not affected. This decrease in slanDC frequency occurred very early during the first week of laquinimod treatment. A saturation effect was found at a laquinimod dose of 1.5 mg such that a further increase of laquinimod dose did not result in additional decreases in slanDC frequency in peripheral blood. Moreover, the decrease in CD83 and TNF expression by the slanDCs following laquinimod treatment suggests a possible mechanism by which laquinimod may exert an anti-inflammatory effect. These results are consistent with previous work by Jolivel et al. [22] showing laquinimod’s modulation of human dendritic cells in in vitro and in vivo preparations. Other MS treatments such as glatiramer acetate [28, 29], fingolimod [30, 31], and fumarate [32] have reported modulation of monocyte and dendritic cells as potential mechanisms for reducing multiple sclerosis pathology.

## Conclusion

Overall, in this study, laquinimod doses up to 2.7 mg were safely administered to patients with RRMS and a significant dose-dependent in vivo effect of laquinimod on the innate immune system was demonstrated.

## Additional file

Additional file 1: Figure S1. Study MS-LAQ-101 flow chart. **Figure S2.** Average plasma concentrations of laquinimod on Day 21 after repeated daily administration. **Figure S3.** Exposure-dose plots of laquinimod after multiple dose administration. **Table S1.** Distribution of study drug termination reasons. **Table S2.** Biochemistry shift analysis to abnormal levels. **Table S3.** Hematology shift analysis. (DOCX 276 kb)

## Abbreviations

AEs: Adverse events
AhR: Aryl hydrocarbon receptor
ALT: Alanine transaminase
AST: Aspartate transaminase
CNS: Central nervous system
CRP: C-reactive protein
DLT: Dose-limiting toxicity
ECGs: Electrocardiograms
ITT: Intent-to-treat analysis set
PBMC: Peripheral blood mononuclear cell
PCS: Potentially clinically significant
PK: Pharmacokinetic
RRMS: Relapsing-remitting multiple sclerosis
SAE: Serious adverse event
SC: Steering committee
SlanDCs: 6-sulpho LacNAc^+^ dendritic cells
TNF-α: Tumor necrosis factor-α
