# Supplementary material for: Safety and in vivo immune assessment of escalating doses of oral laquinimod in patients with RRMS
Source: J Neuroinflammation. 2017 Aug 31;14:172. doi: 10.1186/s12974-017-0945-z (PMC5577769; doi:10.1186/s12974-017-0945-z)
Supplement: Additional file 1: Figure S1. — Study MS-LAQ-101 flow chart. Figure S2. Average plasma concentrations of laquinimod on Day 21 after repeated daily administration. Figure S3. Exposure-dose plots of laquinimod after multiple dose administration. Table S1. Distribution of study drug termination reasons. Table S2. Biochemistry shift analysis to abnormal levels. Table S3. Hematology shift analysis. (DOCX 276 kb) [file 12974_2017_945_MOESM1_ESM.docx]

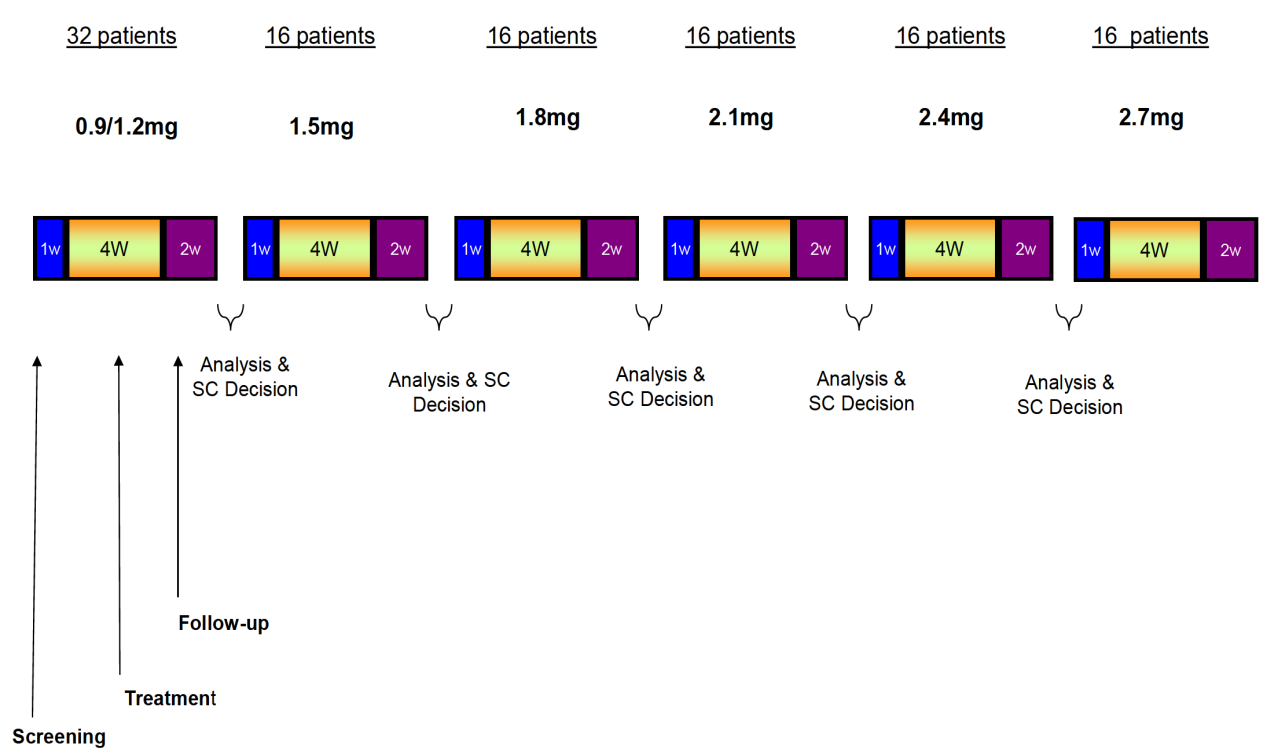


**
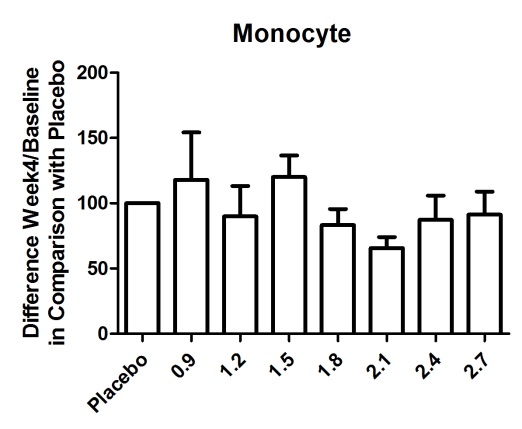

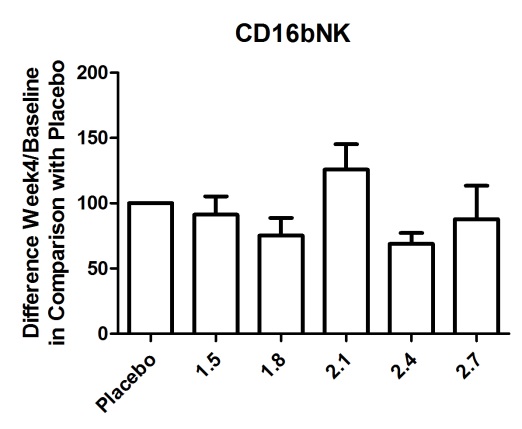

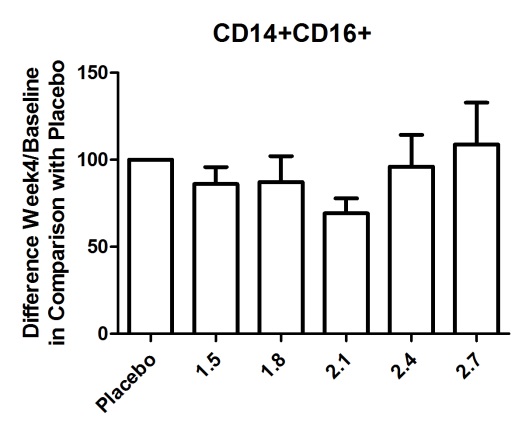
Figure S1. Study MS-LAQ-101 flow chart.**

**SC: Steering Committee; W: week.**

** Figure S2. Average plasma concentrations of laquinimod on Day 21 after repeated daily administration. Samples sizes were n = 10 for the 0.9, 2.4, and 2.7 mg dose groups, n = 12 for the 1.2 and 1.5 mg dose groups, and n = 11 for the 1.8, and 2.1 mg dose groups. Error bars represent standard deviation.**

**Figure S3. Exposure-dose plots of laquinimod after multiple dose administration. Regression line is shown.**

**Table S1: Distribution of study drug termination reasons.**

| **MS-LAQ-101** | | **Pooled Placebo (N=28)** | | **Laquinimod 0.9 mg (N=12)** | | **Laquinimod 1.2 mg (N=12)** | | **Laquinimod 1.5 mg (N=12)** | | **Laquinimod 1.8 mg (N=11)** | | **Laquinimod 2.1 mg (N=13)** | | **Laquinimod 2.4 mg (N=12)** | | **Laquinimod 2.7 mg (N=12)** | | **All** | |
| --- | --- | --- | --- | --- | --- | --- | --- | --- | --- | --- | --- | --- | --- | --- | --- | --- | --- | --- | --- |
|  |  | **N** | **%** | **N** | **%** | **N** | **%** | **N** | **%** | **N** | **%** | **N** | **%** | **N** | **%** | **N** | **%** | **N** | **%** |
| **Randomized** | | 28 | 100.0 | 12 | 100.0 | 12 | 100.0 | 12 | 100.0 | 11 | 100.0 | 13 | 100.0 | 12 | 100.0 | 12 | 100.0 | 112 | 100.0 |
| **Study Drug Termination** | **All** | 27 | 96.4 | 10 | 83.3 | 12 | 100.0 | 12 | 100.0 | 11 | 100.0 | 11 | 84.6 | 11 | 91.7 | 10 | 83.3 | 104 | 92.9 |
|  | **Study Drug Termination** | 27 | 96.4 | 10 | 83.3 | 12 | 100.0 | 12 | 100.0 | 11 | 100.0 | 11 | 84.6 | 11 | 91.7 | 10 | 83.3 | 104 | 92.9 |
| **Prematurely Terminated Study Drug** | **All** | 1 | 3.6 | 2 | 16.7 | . | . | . | . | . | . | 2 | 15.4 | 1 | 8.3 | 2 | 16.7 | 8 | 7.1 |
|  | **Adverse Event** | . | . | 1 | 8.3 | . | . | . | . | . | . | 2 | 15.4 | . | . | 2 | 16.7 | 5 | 4.5 |
|  | **Patient Withdrew Consent** | . | . | . | . | . | . | . | . | . | . | . | . | 1 | 8.3 | . | . | 1 | 0.9 |
|  | **Failed to Return / lost to follow-up** | . | . | 1 | 8.3 | . | . | . | . | . | . | . | . | . | . | . | . | 1 | 0.9 |
|  | **Multiple Sclerosis Relapse** | 1 | 3.6 | . | . | . | . | . | . | . | . | . | . | . | . | . | . | 1 | 0.9 |

**Table S2. Biochemistry shift analysis to abnormal levels.**

| **MS-LAQ-101** | **Pooled Placebo (N=28)** | | **Laquinimod 0.9 mg (N=12)** | | **Laquinimod 1.2 mg (N=12)** | | **Laquinimod 1.5 mg (N=12)** | | **Laquinimod 1.8 mg (N=11)** | | **Laquinimod 2.1 mg (N=13)** | | **Laquinimod 2.4 mg (N=12)** | | **Laquinimod 2.7 mg (N=12)** | |
| --- | --- | --- | --- | --- | --- | --- | --- | --- | --- | --- | --- | --- | --- | --- | --- | --- |
|  | **Change to Low** | **Change to High** | **Change to Low** | **Change to High** | **Change to Low** | **Change to High** | **Change to Low** | **Change to High** | **Change to Low** | **Change to High** | **Change to Low** | **Change to High** | **Change to Low** | **Change to High** | **Change to Low** | **Change to High** |
|  | **%** | **%** | **%** | **%** | **%** | **%** | **%** | **%** | **%** | **%** | **%** | **%** | **%** | **%** | **%** | **%** |
| **Sodium (mmol/L)** | 0 | 10.7 | 0 | 18.2 | 0 | 8.3 | 0 | 16.7 | 18.2 | 0 | 7.7 | 0 | 8.3 | 0 | 25.0 | 0 |
| **Potassium (mmol/L)** | 0 | 7.1 | 0 | 0 | 0 | 25.0 | 8.3 | 16.7 | 0 | 9.1 | 0 | 23.1 | 0 | 0 | 0 | 0 |
| **Calcium (mmol/L)** | 46.4 | 0 | 18.2 | 0 | 66.7 | 0 | 25.0 | 0 | 27.3 | 0 | 23.1 | 0 | 41.7 | 0 | 41.7 | 0 |
| **Phosphorus (mmol/L)** | 0 | 3.6 | 0 | 9.1 | 0 | 8.3 | 8.3 | 0 | 9.1 | 18.2 | 7.7 | 7.7 | 8.3 | 0 | 25.0 | 0 |
| **Glucose (mmol/L)** | 21.4 | 0 | 18.2 | 0 | 8.3 | 0 | 0 | 0 | 0 | 18.2 | 0 | 15.4 | 0 | 8.3 | 8.3 | 8.3 |
| **Creatinine (umol/L)** | 21.4 | 3.6 | 9.1 | 0 | 25.0 | 0 | 25.0 | 0 | 18.2 | 0 | 30.8 | 0 | 41.7 | 0 | 16.7 | 0 |
| **CPK (IU/L)** | 3.6 | 21.4 | 9.1 | 9.1 | 0 | 8.3 | 0 | 16.7 | 9.1 | 9.1 | 7.7 | 7.7 | 8.3 | 8.3 | 0 | 0 |
| **AST (IU/L)** | 0 | 7.1 | 0 | 9.1 | 0 | 8.3 | 0 | 25.0 | 0 | 18.2 | 0 | 23.1 | 0 | 8.3 | 0 | 25.0 |
| **ALT (IU/L)** | 0 | 7.1 | 9.1 | 9.1 | 0 | 16.7 | 0 | 33.3 | 0 | 18.2 | 0 | 23.1 | 0 | 16.7 | 0 | 41.7 |
| **GGT (IU/L)** | 0 | 3.6 | 0 | 0 | 0 | 8.3 | 0 | 33.3 | 0 | 18.2 | 0 | 7.7 | 0 | 8.3 | 0 | 16.7 |
| **Total Bilirubin (umol/L)** | 0 | 0 | 0 | 0 | 0 | 0 | 16.7 | 0 | 9.1 | 0 | 0 | 0 | 8.3 | 0 | 0 | 0 |
| **Total Protein (g/L)** | 14.3 | 0 | 0 | 0 | 8.3 | 0 | 0 | 0 | 9.1 | 0 | 7.7 | 0 | 8.3 | 0 | 16.7 | 0 |
| **Fibrinogen (g/L)** | 3.6 | 14.3 | 0 | 36.4 | 0 | 25.0 | 0 | 16.7 | 0 | 27.3 | 0 | 46.2 | 0 | 33.3 | 0 | 66.7 |
| **C-Reactive Protein (mg/L)** | 0 | 7.1 | 0 | 9.1 | 0 | 25.0 | 0 | 16.7 | 0 | 9.1 | 0 | 30.8 | 0 | 16.7 | 0 | 66.7 |
| **P-Amylase (IU/L)** | 3.6 | 0 | 0 | 0 | 0 | 16.7 | 0 | 25.0 | 0 | 18.2 | 0 | 30.8 | 0 | 33.3 | 0 | 50.0 |

ALT: alanine transaminase; AST: aspartate transaminase; CPK: creatine phosphokinase; GGT: gamma glutamyl transferase.

Incidence of post-baseline shifts to abnormal levels are presented for biochemical parameters with a higher incidence of shifts in any laquinimod dose group compared to the pooled placebo.

**Table S3. Hematology shift analysis.**

| **MS-LAQ-101** | **Pooled Placebo (N=28)** | | **Laquinimod 0.9 mg (N=12)** | | **Laquinimod 1.2 mg (N=12)** | | **Laquinimod 1.5 mg (N=12)** | | **Laquinimod 1.8 mg (N=11)** | | **Laquinimod 2.1 mg (N=13)** | | **Laquinimod 2.4 mg (N=12)** | | **Laquinimod 2.7 mg (N=12)** | |
| --- | --- | --- | --- | --- | --- | --- | --- | --- | --- | --- | --- | --- | --- | --- | --- | --- |
|  | **Change to Low** | **Change to High** | **Change to Low** | **Change to High** | **Change to Low** | **Change to High** | **Change to Low** | **Change to High** | **Change to Low** | **Change to High** | **Change to Low** | **Change to High** | **Change to Low** | **Change to High** | **Change to Low** | **Change to High** |
|  | **%** | **%** | **%** | **%** | **%** | **%** | **%** | **%** | **%** | **%** | **%** | **%** | **%** | **%** | **%** | **%** |
| **WBC (x10^-9^/L)** | 3.6 | 14.3 | 0 | 27.3 | 0 | 8.3 | 0 | 0 | 0 | 0 | 0 | 7.7 | 0 | 8.3 | 0 | 8.3 |
| **Neutrophils Absolute (x10^-9^/L)** | 7.1 | 14.3 | 0 | 27.3 | 0 | 25.0 | 0 | 16.7 | 0 | 0 | 0 | 0 | 0 | 8.3 | 0 | 25.0 |
| **Lymphocytes Absolute (x10^-9^/L)** | 0 | 0 | 9.1 | 0 | 0 | 0 | 0 | 0 | 0 | 0 | 0 | 0 | 8.3 | 0 | 0 | 8.3 |
| **Monocytes Absolute (x10^-9^/L)** | 3.6 | 14.3 | 0 | 27.3 | 0 | 8.3 | 0 | 25.0 | 0 | 9.1 | 0 | 23.1 | 8.3 | 8.3 | 0 | 33.3 |
| **Eosinophils Absolute (x10^-9^/L)** | 3.6 | 3.6 | 0 | 0 | 0 | 0 | 0 | 0 | 0 | 0 | 7.7 | 7.7 | 8.3 | 8.3 | 0 | 0 |
| **Neutrophils (%)** | 7.1 | 10.7 | 0 | 18.2 | 0 | 16.7 | 0 | 0 | 0 | 18.2 | 0 | 15.4 | 0 | 16.7 | 0 | 8.3 |
| **Lymphocytes (%)** | 10.7 | 3.6 | 18.2 | 0 | 25.0 | 0 | 8.3 | 0 | 18.2 | 0 | 15.4 | 0 | 8.3 | 0 | 8.3 | 0 |
| **Monocytes (%)** | 0 | 7.1 | 0 | 0 | 0 | 0 | 8.3 | 0 | 0 | 0 | 0 | 0 | 8.3 | 0 | 0 | 8.3 |
| **Eosinophils (%)** | 0 | 0 | 0 | 0 | 0 | 8.3 | 0 | 0 | 0 | 0 | 0 | 0 | 0 | 0 | 0 | 8.3 |
| **HGB (g/dL)** | 25.0 | 0 | 9.1 | 0 | 25.0 | 0 | 16.7 | 0 | 18.2 | 0 | 38.5 | 0 | 33.3 | 0 | 16.7 | 0 |
| **HCT (%)** | 7.1 | 7.1 | 0 | 0 | 0 | 0 | 8.3 | 0 | 9.1 | 9.1 | 0 | 0 | 0 | 0 | 8.3 | 0 |
| **RBC (x10^-12^/L)** | 14.3 | 10.7 | 0 | 0 | 8.3 | 0 | 16.7 | 0 | 18.2 | 0 | 15.4 | 0 | 33.3 | 0 | 16.7 | 0 |
| **MCV (fL)** | 0 | 14.3 | 0 | 9.1 | 0 | 0 | 0 | 0 | 0 | 9.1 | 0 | 15.4 | 8.3 | 8.3 | 0 | 0 |
| **MCH (pg)** | 0 | 0 | 0 | 0 | 0 | 0 | 0 | 0 | 9.1 | 0 | 7.7 | 0 | 0 | 0 | 0 | 0 |
| **Platelet (x10^-9^/L)** | 7.1 | 0 | 0 | 0 | 0 | 0 | 8.3 | 0 | 18.2 | 0 | 7.7 | 0 | 8.3 | 8.3 | 0 | 0 |

HCT: hematocrit; HGB: hemoglobin; MCH: mean corpuscular hemoglobin; MCV: mean corpuscular volume; RBC: red blood cell (count); WBC: white blood cell (count).

Incidence of post-baseline shifts to abnormal levels are presented for hematological parameters with a higher incidence of shifts in any laquinimod dose group compared to the pooled placebo.

**Preparation of peripheral blood mononuclear cell (PBMC) samples**PBMCs were prepared by Ficoll-Hypaque (Biochrom, Berlin, Germany) density centrifugation. To determine the frequency of 6-sulpho LacNAc+ dendritic cells (slanDCs), PBMCs were incubated with the slan-reactive antibody M‑DC8 followed by fluorescein isothiocyanate (FITC)-conjugated anti‑mouse immunoglobulin M (IgM)-specific goat F(ab)2 (Beckmann Coulter, Marseille, France), peridinin chlorophyll protein complex (PerCP)‑conjugated anti-human leukocyte antigen-antigen D Related (HLA-DR) (BD Biosciences, Heidelberg, Germany), allophycocyanin (APC)‑conjugated anti‑CD14 (BD Biosciences) and phycoerythrin (PE)-conjugated anti-CD16 (BD Biosciences), each for 15 minutes at 4°C. FITC-conjugated anti‑mouse IgM without primary antibody was used as a negative control. Monocytes were characterized by PerCP-conjugated anti‑HLA-DR and APC‑conjugated anti-CD14 (BD Biosciences). Negative controls included directly labeled or unlabeled isotype‑matched irrelevant antibodies (BD Biosciences).

For further characterization of slanDCs and monocytes during relapse treatment, PBMCs were suspended in culture medium consisting of Roswell Park Memorial Institute (RPMI) 1640 (Biochrom), 5% human AB serum (CC pro, Neustadt, Germany), 2 mM L-glutamine, 100 U/mL penicillin, and 100 mg/mL streptomycin (Biochrom). PBMCs (2 x 10^5^ cells/well) were plated on round-bottomed 96‑well plates. After 6 hours, PBMCs were stimulated with 100 ng/mL lipopolysaccharide (LPS) (Sigma–Aldrich) or 10 mM R848 (InvivoGen, Toulouse, France) in the presence of 0.2 mM Monensin (Biomol, Hamburg, Germany) for tumor necrosis factor (TNF)-α staining and harvested 12 hours later. Intracellular molecules were investigated by cell fixation with ice-cold 4% paraformaldehyde (Merck) for 15 minutes, and permeabilized with 0.1% saponin (Merck) in phosphate-buffered saline (PBS; Biochrom) plus 1% fetal calf serum (FCS; Biochrom) for 3 minutes at 4°C. Afterwards, cells were incubated with a PE-conjugated anti-TNF-α or PE-conjugated isotype‑matched irrelevant antibody (BD Biosciences) for 15 minutes. Cells were evaluated on a fluorescence-activated cell sorting (FACS)‑Calibur cytometer (BD Biosciences).
